# Antimicrobial Susceptibility Profiles of Commensal *Enterococcus* spp. Isolates from Chickens in Hungarian Poultry Farms Between 2022 and 2023

**DOI:** 10.3390/antibiotics13121194

**Published:** 2024-12-07

**Authors:** Ádám Kerek, Ábel Szabó, Ákos Jerzsele

**Affiliations:** 1 Department of Pharmacology and Toxicology, University of Veterinary Medicine, István utca 2, 1078 Budapest, Hungary; szabo.abel@student.univet.hu (Á.S.); jerzsele.akos@univet.hu (Á.J.); 2 National Laboratory of Infectious Animal Diseases, Antimicrobial Resistance, Veterinary Public Health and Food Chain Safety, University of Veterinary Medicine, 1078 Budapest, Hungary

**Keywords:** *Enterococcus*, antimicrobial resistance, minimum inhibitory concentration, MIC, poultry, chickens, Hungary

## Abstract

**Background:** The global spread of antimicrobial resistance (AMR) represents one of the most significant challenges of our generation. It is crucial to continuously monitor AMR, not only by investigating clinical, pathogenic strains but also by monitoring commensal bacterial strains, as they can serve as natural reservoirs of resistance. Infections caused by *Enterococcus* species are increasingly recognized as emerging threats to both animal and public health. Among economically important livestock, poultry as a major source of animal protein for humans is a frequent carrier of enterococci, and also of sporadically detected clinical disease. **Methods:** This study aimed to determine the antimicrobial susceptibility profile of *Enterococcus* strains (*n* = 499) isolated from chicken farms in Hungary. The minimum inhibitory concentration (MIC) was determined for 15 antibiotics, including 10 with established clinical breakpoints. **Results:** The strains exhibited good sensitivity to amoxicillin, one of the first-line treatments for *Enterococcus* infections in veterinary medicine, with only 20.8% showing resistance. However, we observed an alarming 27.9% resistance rate to vancomycin, which is reserved to treat infections caused by multidrug-resistant strains in humans. A comparison of our findings with Hungarian hospital records revealed that the resistance patterns of poultry-derived *Enterococcus faecalis* strains were very similar to those of human isolates, particularly regarding penicillins and aminoglycosides. **Conclusions:** Overall, the increasing rates of AMR reinforce the importance of conducting periodic studies to establish long-term trends. For multidrug-resistant strains, next-generation sequencing is recommended to elucidate the genetic basis of resistance.

## 1. Introduction

Antimicrobial resistance (AMR) is a phenomenon where microorganisms develop resistance to specific antimicrobial agents, posing serious health risks [1]. According to conservative estimates, AMR is projected to be responsible for 10 million deaths annually by 2050 [2]. Currently, the European Union (EU) reports that over 670,000 infections are linked to AMR each year, resulting in nearly 33,000 deaths directly attributable to confirmed resistance [3].

Poultry farming is one of the leading sectors in global animal protein production, owing to the relatively low production costs and the fact that poultry meat consumption is neither restricted nor prohibited by any major religion [3]. In the EU alone, approximately fifteen million tons of poultry meat and over seven million tons of eggs are produced annually [4].

In poultry, the most commonly identified facultative pathogens include *Escherichia coli*, *Campylobacter* spp., *Enterococcus faecalis* (*E. faecalis*), *Enterococcus faecium* (*E. faecium*), *Enterococcus durans*, *Enterococcus avium*, *Enterococcus hirae*, and *Enterococcus cecorum*. These species are part of the normal gut microbiome in chickens but are increasingly isolated from broilers suffering from conditions such as spondylitis, osteomyelitis, arthritis, and pericarditis, often due to predisposing factors (stress, nutritional imbalance, etc.) [5]. *E. faecalis* infections can affect poultry across various species and ages, but they are particularly significant in embryonic and young birds. This pathogen plays a dominant role in the gut microbiome of day-old chicks, who are especially vulnerable during the first week of life, manifesting as sepsis (with high mortality), umbilicitis, pericarditis, or salpingitis [6,7]. In older birds, infections may lead to arthritis and amyloidosis [8].

Among the resistance mechanisms in *Enterococcus* species, the modification of binding sites is particularly noteworthy. A typical example is the resistance developed against glycopeptide agents, where the precursor of the peptidoglycan structure, acyl-D-alanyl-D-alanine, is replaced by D-Ala-D-Lac or D-Ala-D-Ser. This process is governed by a complex gene cluster (*vanA-N*) [9]. *Enterococcus* species are intrinsically resistant to cephalosporins and exhibit natural reduced sensitivity to penicillins due to the expression of low-affinity penicillin-binding proteins (PBPs), which weakly bind to beta-lactam antibiotics [10]. Additionally, *Enterococcus* species are intrinsically resistant to clinically achievable concentrations of aminoglycosides because of their low cell wall permeability [11]. They are also intrinsically resistant to clindamycin and lincomycin [12].

The role of vancomycin-resistant *Enterococcus* strains in nosocomial infections (hospital-acquired infections) led to the European Commission’s 1997 decision to ban avoparcin as a growth promoter. Later, in 2006, the emergence of vancomycin resistance prompted a ban on all antimicrobial growth promoters within the EU [13]. These restrictions, combined with the increasing intensity of poultry farming, may have contributed to the rising incidence of bacterial infections in the sector, with *Enterococcus* strains playing a significant role [14]. The overuse and misuse of antibiotics, particularly as growth promoters in livestock farming in non-EU countries, including in poultry farming, is a major driver of the increasing spread of AMR [4]. The economic impact of resistant microorganisms in the gut microbiome and their potential transmission to humans is also a significant concern [15].

The global spread of vancomycin-resistant *Enterococcus* (VRE) strains is particularly alarming. The European Antimicrobial Resistance Surveillance System (EARSS) reported an increase in the prevalence of clinical vancomycin-resistant *E. faecium* in Europe, from 10.5% in 2015 to 17.3% in 2018 [16]. Vancomycin resistance in *Enterococcus* species is linked to the introduction of avoparcin, a glycopeptide antibiotic, as a growth promoter in 1975 [17], which was extensively used in broilers and turkeys in the past [18,19]. This practice led to cross-resistance to vancomycin, as evidenced by the steady rise in VRE strains [20]. In countries like the United States, where avoparcin was not authorized, no VRE strains were isolated from farm animals until 2008. After the ban, the percentage of VRE strains began to decline, but they did not disappear completely. This persistence might be due to the co-selection of resistance genes by tylosin, commonly used in pigs and poultry, which often harbor resistance genes for both drugs on the same plasmid [21].

The prevalence of multidrug-resistant (MDR) *Enterococcus* strains is increasing in nosocomial infections [22]. Following antibiotic treatment, these strains rapidly colonize the gastrointestinal tract, outcompeting the commensal bacteria that constitute a healthy gut microbiome [23,24]. The widespread use of antibiotics has contributed to the nosocomial spread of resistant *Enterococcus* strains to such an extent that these bacteria have become one of the most common infectious agents in hospital settings. The ability of *Enterococcus* species to survive outside the gastrointestinal tract on various surfaces and medical equipment further facilitates their spread, with hospital staff potentially transmitting the pathogen directly or indirectly between patients [22]. To reduce selection pressure from antibiotic use, alternatives such as antimicrobial peptides [25], essential oils [26], and various plant extracts [27,28] have been suggested. Propolis, in particular, shows promise as an effective alternative, especially in pigs [29,30,31] and poultry [32], which consume the largest quantities of antibiotics [33]. Furthermore, the appropriate application of the antimicrobial substances also contributes greatly to reducing the level of resistance; pharmacokinetic/pharmacodynamic (PK/PD) model studies are crucial for selecting appropriate therapies [34].

AMR is a global challenge requiring a multifaceted approach to ensure the sustainability of effective antibiotics. Collective thinking, cooperation, and responsible action are essential to effectively reduce AMR and the incidence of infections caused by MDR pathogens. Collaborative efforts between the veterinary and public health sectors, along with stricter regulations, guidelines on antibiotic use, and increased awareness, are vital to managing and mitigating AMR in the future. Regular surveillance studies, such as our research, provide valuable insights not only for animal health but also for public health, reinforcing the One Health principle through mutual understanding.

## 2. Results

### 2.1. Regional Distribution and Origin of the Samples

**Figure 1:**
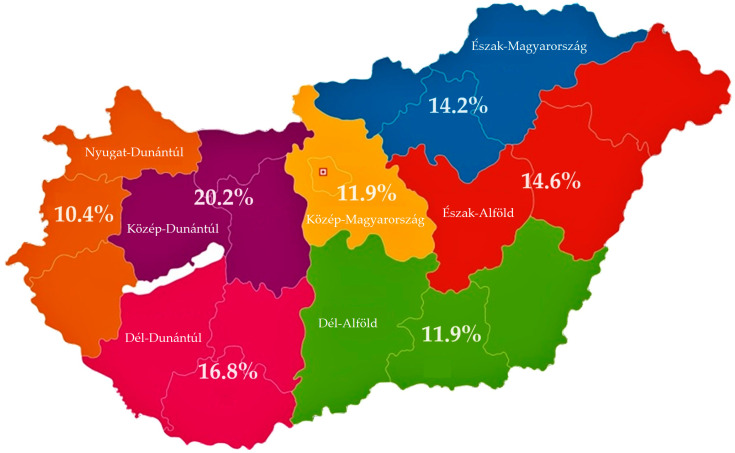
The geographical distribution of the *Enterococcus* samples (*n* = 499) in Hungary.

A total of 499 *Enterococcus* isolates were tested against 15 antibiotics that are significant in veterinary and public health. The prevalence, based on the number of samples and isolates, is 72.32%. The 95% confidence interval (CI) is between 68.86% and 75.53%, calculated from the 690 samples analyzed. The samples originated from a total of 23 livestock farms, ensuring nationwide coverage, with at least 3 farms represented from each region to strive for nearly representative sampling (Supplementary Table S1) between 2022 and 2023. At each farm, 15 oral–pharyngeal swab samples and 15 cloacal swab samples were collected and sent to the reference laboratory for isolation. This totaled 345 animals, as both oral–pharyngeal and cloacal swab samples were collected from the same animals by the attending veterinarians. Figure 1 shows the geographical distribution of the isolates.

### 2.2. Antimicrobial Susceptibility Testing

**Figure 2:**
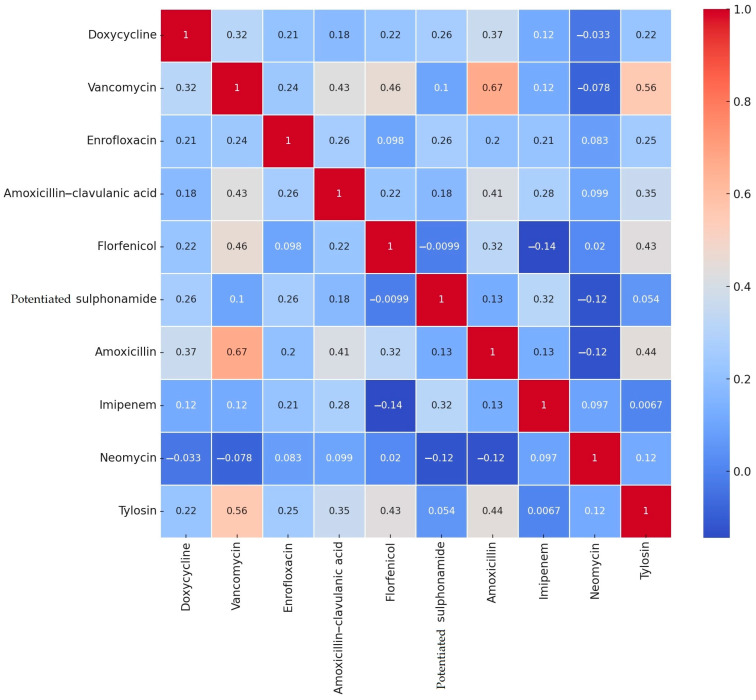
Correlation heat map of the degree of drug resistance of *Enterococcus* samples (*n* = 499) isolated from chicken farms.

Of the active substances tested, ten had established breakpoints. The resistance rates for each strain and the correlation values between individual antibiotics are presented in a heat map (Figure 2). A positive correlation (+1) indicates that the resistance patterns of the antibiotics are similar. A correlation close to 0 suggests no relationship between antimicrobial resistance patterns. Strong positive correlations were observed, such as between amoxicillin and vancomycin (0.67) and between tylosin and vancomycin (0.56). Negative correlations are indicated by negative values, with the weakest negative correlations observed between imipenem and florfenicol (−0.14), neomycin and potentiated sulfonamides (−0.12), and amoxicillin and neomycin (−0.12). The *p*-values for the correlation test results are reported in Supplementary Table S2.

We compared the resistance results based on the sample source (respiratory vs. cloacal) to determine if there were differences in the degree of resistance (Table 1). The data did not show a normal distribution, so the Mann–Whitney U-test was used when comparing two groups, and the Kruskal–Wallis H-test was used when comparing more than two groups. Significant differences were identified for amoxicillin (*p* = 0.0421) and neomycin (*p* = 0.0001), indicating a significant variation in resistance patterns for these antibiotics between the two sample sources.

We also compared the resistance rates for different types of chickens (laying, meat, breeding) to determine if there were significant differences (Table 2). Significant differences were observed in most cases, indicating that the type of chicken influences the resistance to various antibiotic active substances. However, resistance to doxycycline did not significantly differ based on the utilization. Additionally, the differences in resistance between laying and breeding flocks were not significant for enrofloxacin, amoxicillin, neomycin, or tylosin. Similarly, between laying and meat flocks, no significant differences were found for potentiated sulfonamides and imipenem. The largest differences were observed between meat and breeding flocks, where doxycycline was the only antibiotic for which no significant difference in resistance was detected.

We investigated whether there was a significant difference in resistance levels based on age (Table 3). Significant differences were observed in most cases, except for doxycycline and imipenem, where no significant differences were found.

We also examined the relationship between flock size and antimicrobial resistance by active substance (Table 4). Resistance patterns were generally less dependent on flock size. However, significant differences were found between small and medium flocks for enrofloxacin, neomycin, and tylosin. Between small and large flocks, significant differences were observed for doxycycline, potentiated sulfonamide, neomycin, and tylosin. For medium and large flocks, significant differences were noted for doxycycline, enrofloxacin, potentiated sulfonamide, and neomycin.

**Figure 3:**
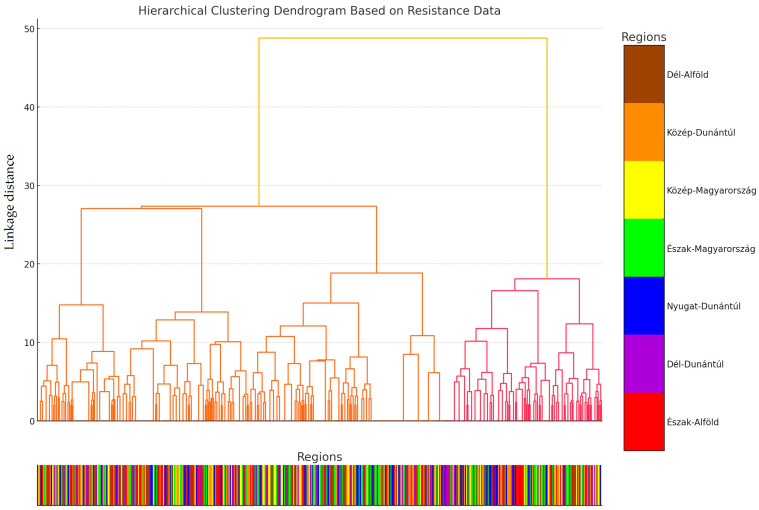
Cluster analysis plotted as a dendrogram by strain of the antimicrobial resistance profile according to the distance of each cluster. To maintain clarity, we did not label the identity numbers of each sample on the horizontal axis. Instead, we categorized each sample by its region of origin and color-coded the horizontal axis lines accordingly.

We analyzed the degree of drug resistance for each strain and then used hierarchical cluster analysis to plot the strains on a dendrogram (Figure 3). The vertical axis displays the distance values, representing the differences between the various clusters, while the lines on the dendrogram illustrate the clustering hierarchy. Individual lines connect patterns or clusters that are grouped based on similarity. The lower the line, the more similar the connected elements are. By intersecting the lines on the dendrogram, the number of clusters can be determined; for example, by drawing a horizontal line across the dendrogram at a certain height, the number of intersected lines indicates the number of clusters.

**Figure 4:**
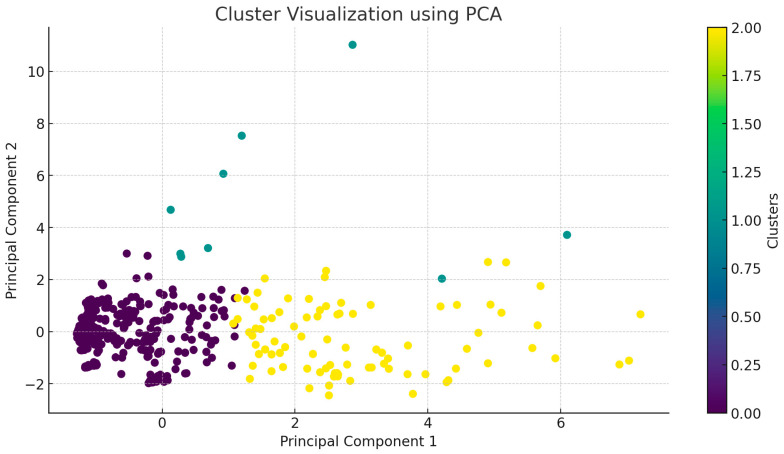
After principal component analysis, the data were grouped into three clusters (purple—Cluster 0, green—Cluster 1, yellow—Cluster 2).

The cluster analysis identified a total of three main clusters: Cluster 0 contained 403 samples, Cluster 1 had 9 samples, and Cluster 2 included 87 samples. We then conducted a principal component analysis (PCA) and plotted the data (Figure 4), with each cluster represented by a different color. To select the three main clusters, we performed the elbow method, silhouette analysis, and gap statistics. The elbow method (Supplementary Figure S1) indicated a significant slowdown in the decrease in the within-cluster sum of squares at three clusters. The silhouette analysis (Supplementary Figure S2) showed that the silhouette score was maximized for three clusters, indicating better grouping with higher scores. Similarly, the gap statistic (Supplementary Figure S3) graphically demonstrated that the value was also maximized at three clusters. Based on these analyses, the optimal choice was three clusters.

The two main axes in the figure, Principal Component 1 and Principal Component 2, represent the directions that account for the largest variance in the data. These axes form a new coordinate system that allows the visualization of the data in two dimensions while retaining as much information as possible. Each point in the figure represents a bacterial sample from the database, characterized by its antimicrobial resistance data. The figure illustrates how the three identified clusters are positioned relative to each other in the principal component space, with each cluster reflecting different antimicrobial resistance patterns. Cluster 0 (in purple), which contains significantly more samples than the other two clusters, is the largest. Cluster 2 (in yellow) is medium-sized, while Cluster 1 (in green) is the smallest. The principal components capture most of the variance, so the distribution of points along these axes reflects the variability in antimicrobial resistance patterns. Points that are more dispersed along the principal components indicate greater differences in resistance patterns.

Notably, samples in Cluster 1 originated exclusively from the “Észak-Alföld” and “Észak-Magyarország” regions. In contrast, samples in Cluster 0 were evenly distributed across all regions. For Cluster 2, the highest frequency of patterns was observed in the “Dél-Dunántúl” and “Közép-Dunántúl” regions, with no patterns identified in the “Nyugat-Dunántúl” or “Észak-Magyarország” regions.

For each active substance, the MIC values were determined for each strain to create a frequency table of their distribution and the percentage of samples (Table 5). Additionally, the MIC_50_ and MIC_90_ values for each active substance were calculated. The ECOFF values provided by EUCAST were also plotted against the data. The calculated MIC_50_ and MIC_90_ values were below the CLSI-defined breakpoints for imipenem. For neomycin, doxycycline, florfenicol, vancomycin, amoxicillin, amoxicillin–clavulanic acid, and enrofloxacin, only the MIC_50_ values were below the breakpoints. Compared to the ECOFF values, the MIC_50_ values for florfenicol and vancomycin exceeded the thresholds.

We also assessed the susceptibility of agents for which no breakpoints are available, those that are impractical for use in *Enterococcus* infections (such as spectinomycin, tiamulin, and lincomycin), or those generally ineffective against *Enterococcus* due to specific mechanisms, like ceftriaxone (low affinity for penicillin-binding proteins) or colistin (different mechanism of action). The frequency table, along with MIC_50_ and MIC_90_ values for these agents, is summarized in Supplementary Table S3.

**Figure 5:**
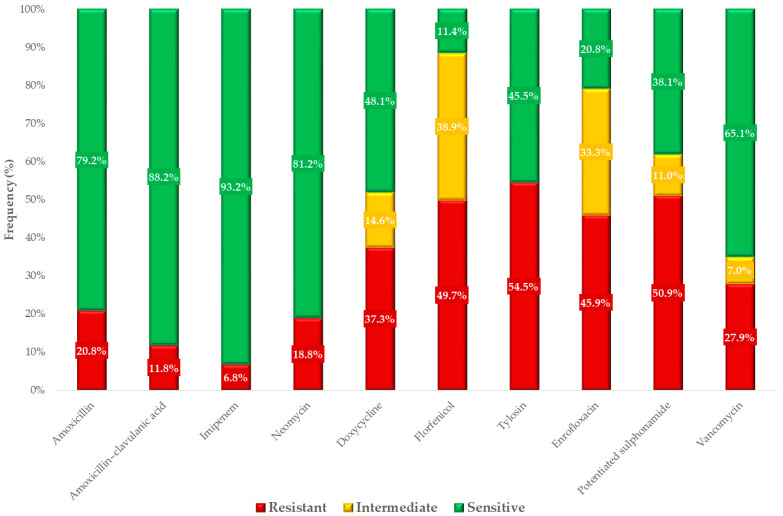
Susceptibility profile of *Enterococcus* strains (*n* = 499) isolated from chickens to antibiotic agents of animal and public health importance.

Breakpoints for a total of 10 different active substances were used to determine the sensitivity profile of the strains for each substance (Figure 5).

**Figure 6:**
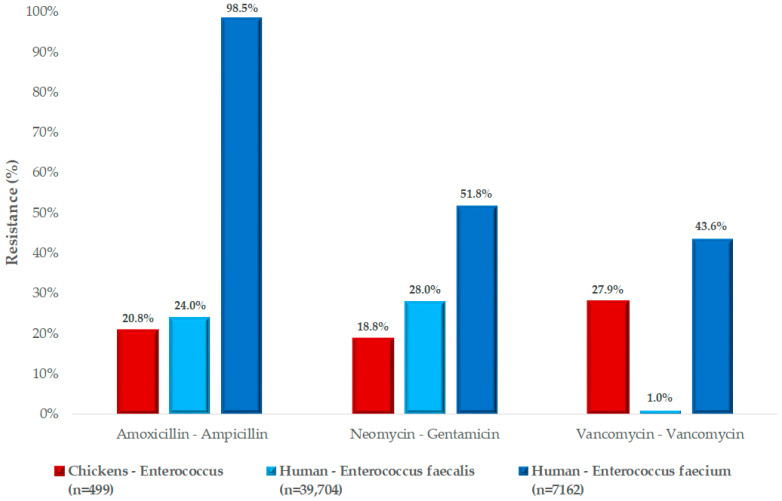
Susceptibility profiles of human and poultry isolates based on a comparison of available active substances.

We had the opportunity to compare our results with human resistance data (Figure 6). Among the available data, amoxicillin is widely used and authorized in veterinary medicine, while in public health, ampicillin is the preferred equivalent. Amoxicillin–clavulanic acid and imipenem are also used in human medicine, due to a penicillin-binding protein insertion site mutation specific to *Enterococcus* species. In our tests, we observed resistance rates of 20.8% for amoxicillin in samples from chickens. Generally, human *E. faecalis* strains were more sensitive (24.0%) than *E. faecium* strains (98.5%). However, since species-level separation was not possible, our results are presented at the genus level. Our results for amoxicillin were consistent with those observed for *E. faecalis* in humans.

For aminoglycosides, we compared human results with neomycin, which is the approved alternative for use in poultry. The resistance level to neomycin in our study was similar (18.8%) to that of human *E. faecalis* strains (28.0%) to gentamicin.

Regarding vancomycin, in this experiment, the resistance levels in animal health were found to be very similar to the average resistance levels observed in human cases.

## 3. Discussion

A total of 499 *Enterococcus* strains were tested to assess their susceptibility to active substances of importance in both animal and public health on a national level. Our results were largely consistent with those of other international studies. However, one of the limitations of our research lies in the differences in antibiotic use between countries, which can vary significantly even between individual livestock farms. These differences in selective pressure can lead to varying resistance patterns.

We aimed to ensure a nearly representative sampling that covered all regions. While we isolated a substantial number of strains, it is important to note that a larger sample size provides a more accurate and realistic picture. Despite the extensive effort involved in sample collection and analysis over more than a year, the most significant limitation to further increasing the sample size was human resources.

Another limitation in comparing our findings with human resistance data is the scale, as human data were derived from a sample size several orders of magnitude larger. The results would gain greater clarity in the future if we could also determine the resistance profiles of strains isolated from clinical cases.

For amoxicillin, Bekele et al. reported higher resistance rates of 43–63% in samples from chickens and cattle in Ethiopia [35], and Ayeni et al. found a much higher resistance rate of 73.3% in chickens in Nigeria [36]. However, Lemsaddek et al. observed lower levels of resistance to penicillin, at 33.3% and 6.25% for chickens housed in conventional and free-range systems, respectively [37]. In comparison, we found resistance to amoxicillin in 20.8% of strains. Although amoxicillin–clavulanic acid is not approved for use in poultry, we tested for its public health relevance, revealing a resistance rate of 11.8%, almost identical to that of amoxicillin, which supports the fact that *Enterococcus* strains do not produce β-lactamase. Notably, Pesavento et al. reported a resistance rate of only 1.47% to *Enterococcus* strains in human foodstuffs [38]. The similar resistance levels of amoxicillin and amoxicillin–clavulanic acid support that *Enterococcus* strains are not characterized by β-lactamase production. For imipenem, our study observed a resistance rate of 6.8%, while Schwaiger et al. reported 4.3% resistance in organic chicken farming and 5.5% in conventional chicken farming [39], Li et al. found a resistance rate of 15.9% [40], and Roy et al. described an even higher resistance rate of 55.6% [41]. In summary, we can conclude that the β-lactam agents used in the treatment of infections caused by *Enterococcus* species have retained their efficacy; the limitation in studies involving imipenem may be its instability in aqueous solutions, as reported in other publications.

In our studies, resistance to neomycin was observed in 18.8% of strains, whereas Lanza et al. reported a significantly higher resistance rate of 83% [42]. Doxycycline resistance was observed in 37.3% of strains in our study. Bekele et al. found resistance ranging from 26 to 41% in different colonies [35], while Schwaiger et al. observed a resistance rate of 39.7% in organic farming and 70.1% in conventional farming [39]; Noh et al. reported that 58.2% of strains were resistant [43]. This shows that there is considerable variation between countries, depending on antibiotic use and husbandry practices, but the type of utilization can also be a factor.

Florfenicol resistance accounted for 49.7% of the strains in our study, whereas Schwaiger et al. did not detect any resistant strains in either organic or conventional farming [39], Karunarathna et al. found resistance in only 0.4% of strains [44], and Kim et al. reported resistance rates between 14.3% and 18.7% [45]. Tylosin resistance was observed in 54.5% of our strains, a result similar to the values reported by Kim et al., which ranged between 53% and 63.6% [45]. The decreased sensitivity to these agents is likely due to excessive or widespread use, although they are generally not the first-choice drugs for treating *Enterococcus* infections.

Enrofloxacin, a widely used active substance in the poultry sector, showed a resistance rate of 45.9% in our study. Oliveira et al. observed a much higher resistance rate of 83.3% [46], while Karunarathna et al. reported only 5.1% resistance [44], and Liu et al. found a resistance rate of 29.6% [47]. The 45.9% resistance to enrofloxacin observed in commensal *Enterococcus* strains from poultry may indicate that this antibiotic is likely used widely in the poultry industry, potentially not only for therapeutic purposes but possibly also as a preventative measure. This resistance level highlights the probable need to consider limiting enrofloxacin use in poultry and to investigate the likely causes behind the emergence of resistance.

Resistance to potentiated sulfonamide (trimethoprim–sulfamethoxazole in a 1:19 ratio) was 50.9% in our study. Makarov et al. reported resistance rates of 32.9–36.6% [48], while Karunarathna et al. observed a much lower resistance rate of 7.4% [44]. The 50.9% resistance observed to sulfonamides in these strains may reflect the extensive history and prolonged use of this antibiotic class, one of the oldest in veterinary and human medicine. This likely contributed to the selection pressure favoring resistant strains.

Vancomycin resistance was found to be 27.9% in our study. In contrast, Schwaiger et al. [39] and Semedo-Lemsaddek et al. [37] did not detect any resistance. Karunarathna et al. reported only 1.9% resistance [44], Pesavento et al. found 2.9% [38], Roy et al. reported 4.4% [41], Bekele et al. observed resistance ranging from 15 to 66% in different colonies [35], Ayeni et al. found resistance as high as 65% [36], and Liu et al. observed resistance levels exceeding 92% [47]. Vancomycin resistance at 27.9% is particularly concerning, especially given that vancomycin is a last-line antibiotic reserved for critical cases in human medicine. This high level of resistance may indicate a troubling trend, as it likely reduces the effectiveness of an essential therapeutic option and might facilitate the horizontal transfer of resistance genes, which could impact treatment outcomes for severe infections.

In the poultry sector, the emergence of resistant bacterial strains, particularly *Enterococcus* species, shows a concerning trend that is likely a consequence of widespread antibiotic use. The results of the studies suggest that the decreasing sensitivity and increasing resistance may lead to a narrowing of treatment options for poultry diseases. Urgent measures are needed to reduce resistance for the sustainability of the poultry industry and the protection of public health, including targeted antibiotic use and stricter animal health regulations.

Among the different grouping criteria, the most significant factor was the age of the animals, where we found a substantial difference in resistance to the active substances in most cases, in favor of older animals. This may be due to the use of different types of antibiotics according to age, and the greater the age of the animal, the more likely it is to encounter more antibiotics. This was followed by the type of utilization, with the most notable differences observed between meat and breeding flocks, where only doxycycline showed no significant difference. In most cases, the level of resistance was significantly higher in meat-producing populations, except in the case of the potentiated sulfonamide, where resistance was significantly greater in breeding stock. Our previous studies on utilization types have shown that the most severe resistance occurs in meat flocks [49]. In this case, the length of the food safety withdrawal period may also be a determining factor, which is taken into account when selecting the antibiotic. Flock size had a much smaller impact, and the source of the sample (trachea or cloaca) was the least significant factor. The latter may be explained by the chickens’ scraping and pecking behavior, which involves ingesting *Enterococcus* species shed in feces that contaminate the environment and subsequently colonize the tracheal mucosa. Another possible explanation is that antibiotics are administered through drinking water, exposing the oral pharyngeal microbiome to the active substances, which are then passed into the intestinal tract.

We compared our results with the available human data. For amoxicillin, resistance rates in *E. faecalis* were very similar for strains isolated from poultry and humans, whereas human *E. faecium* strains exhibited much higher resistance (98.5%). A study conducted in Sweden found that human *E. faecium* samples had a remarkably high resistance rate of 77% for amoxicillin [50], while a Canadian study reported 22% resistance in *E. faecium* strains and 2.9% resistance in *E. faecalis* strains [51]. Our study found a resistance rate of 27.9% to vancomycin. In comparison, Billström et al. reported that 5% of *E. faecium* strains were resistant to vancomycin [51]. Louie et al. found that 22% of *E. faecium* strains were moderately susceptible, with no fully resistant strains detected [51], and Nayak et al. reported that all *Enterococcus* strains tested were susceptible [52].

Among the aminoglycosides, our study found 18.8% resistance to neomycin, while resistance to gentamicin was 28% in human *E. faecalis* strains and 51.8% in human *E. faecium* strains. Billström et al. found that 2% of *E. faecium* strains were resistant [50], whereas Taji et al. reported a 50.9% resistance rate in *E. faecalis* strains [53]. The limitation of our study is that our objective was to represent the level of resistance to the individual antibiotics at the genus level during the investigated time interval. Significant differences between *E. faecalis* and *E. faecium* strains observed in human studies encourage us to conduct species-level separation in the future. Furthermore, we aim to perform additional investigations on multi-resistant strains, particularly those resistant to vancomycin, utilizing next-generation sequencing to further explore the genetic background of resistance. We also intend to investigate the carrying of individual resistance genes on plasmids or phages, as well as their presence as mobile genetic elements. By regularly comparing our results with human data, we strive to increasingly establish the One Health approach and comprehensively monitor the situation regarding resistance.

## 4. Materials and Methods

### 4.1. Origin of Strains and Human Data

The strains analyzed in this study were collected between 2022 and 2023 during routine diagnostic testing conducted by veterinarians working with large animal holdings. The samples were collected by the working veterinarians using sterile Amies-type swabs without charcoal and with standard aluminum shafts (Biolab Zrt., Budapest, Hungary). In each case, two samples were taken from the same animal: one oral–pharyngeal sample from the vicinity of the tracheal entrance and one cloacal swab. Sampling was performed by rotating the swab 3–5 times in a circular motion. Subsequently, the transport media samples were shipped to the reference laboratory under conditions maintained at 2–8 °C, from where we received the samples for further analysis. We spread the samples on m-*Enterococcus* modified agar (Merck KGaA, Darmstadt, Germany) for the identification of the *Enterococcus* genus, following the manufacturer’s specifications, which indicate that only *Enterococcus* species exhibit growth on this medium, with colony colors ranging from light pink to red. The strains transferred to the subculture medium were stored at −80 °C using the Microbank™ system (Pro-Lab Diagnostics, Richmond Hill, ON, Canada) for further processing. Human resistance data were provided by the National Public Health and Pharmaceutical Center. The aggregated and region-specific human resistance data were obtained in an Excel file with the permission of the National Chief Medical Officer. The file contained resistance percentages.

For each sample, detailed information was recorded, including the organ of origin (trachea, cloaca), the location of collection, the type of utilization (meat, eggs, breeding), the age of the animal (young, adult), and the size of the flock (5001–50,000; 50,001–100,000; >100,001). Based on the collection locations, the samples were categorized into seven administrative regions of Hungary.

### 4.2. Minimum Inhibitory Concentration (MIC) Determination

The phenotypic expression of resistance was assessed by determining the minimum inhibitory concentration (MIC) values following the methodology outlined by the Clinical Laboratory Standards Institute (CLSI) [54]. Breakpoints were defined according to CLSI guidelines [54], and the epidemiological cut-off value (ECOFF) was established based on the standards set by the European Committee on Antimicrobial Susceptibility Testing (EUCAST). For neomycin, a breakpoint of 1024 µg/mL was referenced from a meat-products study specific to *Enterococcus* species [55]. Similarly, a breakpoint of 8 µg/mL for tylosin was identified, based on the literature sources [56].

Bacterial strains stored at −80 °C were re-suspended in 3 mL of cation-adjusted Müller–Hinton broth (CAMHB) the day before testing and incubated for 18–24 h at 37 °C. The MIC studies were conducted using 96-well microtiter plates (VWR International, LLC., Debrecen, Hungary). Except for the first column, all wells of the working plates were filled with 90 µL of CAMHB. A stock solution of 1024 µg/mL for the test substances (Merck KGaA, Darmstadt, Germany) was prepared according to CLSI guidelines [54]. The active ingredients amoxicillin and amoxicillin–clavulanic acid in a 2:1 ratio (pH 7.2, 0.01 mol/L) and imipenem (pH 6, 0.1 mol/L) were dissolved in phosphate buffer solution. Doxycycline, neomycin, tylosin, and vancomycin were dissolved in distilled water. For the preparation of the potentiated sulfonamide (trimethoprim and sulfamethoxazole at a 1:19 ratio), sulfamethoxazole was dissolved in hot water with a few drops of 2.5 mol/L NaOH, while trimethoprim was dissolved in distilled water with 0.05 mol/L HCl. Enrofloxacin was prepared using a few drops of 1 mol/L NaOH solution in distilled water. Florfenicol was dissolved using a few drops of 95% ethanol and distilled water.

This stock solution was serially diluted 1:2 with CAMHB broth, starting with 512 µg/mL, and 180 µL of this solution was added to the first column of the working plates to create a 2-fold dilution series. After column 10, the excess 90 µL solution was discarded, leaving 90 µL in each well. Bacterial suspensions were prepared using a nephelometer (ThermoFisher Scientific, Budapest, Hungary) set to 0.5 McFarland, and 10 µL of the suspension was inoculated into each well starting from column 11 of the microtiter plates [54]. The results were evaluated using the Sensititre™ SWIN™ automatic MIC reader (ThermoFisher Scientific, Budapest, Hungary) and VIZION system software version 3.4 (ThermoFisher Scientific, Budapest, Hungary, 2024). *E. faecalis* (ATCC 29212) was used as the reference isolate.

Statistical analysis was performed using R version 4.1.0 [57]. The normality of the data distribution was tested with the Shapiro–Wilk test. Data that did not follow a normal distribution were further analyzed using non-parametric tests. The resistance of each active substance was examined using the Kruskal–Wallis test [58], which does not assume a normal distribution and is suitable for comparing the medians of several sample groups—making it ideal for analyzing differences across various samples. A post hoc test was employed to determine specific correlations between groups. Pairwise comparisons were conducted using the Mann–Whitney U-test [59] and *t*-tests, with Bonferroni correction applied to adjust for inflated *p*-values resulting from multiple comparisons [60]. It is important to note that while the Bonferroni correction reduces the likelihood of Type I errors, it may increase the risk of Type II errors (failure to detect true differences). Further correlation analyses were conducted to explore relationships between individual active substances, followed by principal component analysis (PCA) [61] to identify similarities or differences in patterns. Hierarchical cluster analysis was then performed, with results presented in a dendrogram [62], providing a visual representation of the distances between samples and the clustering hierarchy.

## 5. Conclusions

Our findings align with the existing literature in several key areas, yet they also underscore the significant impact that local and regional antibiotic use patterns have on antimicrobial resistance. Penicillins, such as amoxicillin, which are primarily used to treat *Enterococcus* infections, largely retained their effectiveness, with 20.8% of strains showing resistance. The relatively low resistance to imipenem, a drug reserved for public health use, is consistent with expectations for this bacterial species. However, the relatively high resistance to vancomycin is particularly concerning and highlights the ongoing challenges posed by antimicrobial resistance, as it is a widely relied-upon antibiotic in human medicine.

The increasing prevalence of resistance can be directly linked to patterns of antibiotic usage, emphasizing the need for comprehensive and representative surveillance. Continuous monitoring of resistance levels is crucial, as it allows for the identification of emerging trends and the development of timely, informed interventions.

Future research should focus on strains resistant to antibiotics of public health significance. Utilizing next-generation sequencing to precisely map the genetic mechanisms underlying phenotypic resistance will further enhance our understanding and ability to combat this growing threat.

## Figures and Tables

**Table 1:** Statistical analysis between sampling source and resistance rate.

Active Substance	Respiratory—Cloacal Comparison
	*p*-Values
Doxycycline	0.7157
Vancomycin	0.7283
Enrofloxacin	0.9007
^1^ Amoxicillin–clavulanic acid	0.1171
Florfenicol	0.3437
^2^ Potentiated sulfonamide	0.6073
Amoxicillin	0.0421 *
Imipenem	0.2099
Neomycin	<0.0001 *
Tylosin	0.8249

* Significant difference (*p* < 0.05); ^1^ 1:2 ratio; ^2^ trimetoprim–sulphametoxazole 1:19 ratio.

**Table 2:** Statistical analysis of resistance by type of utilization.

Active Substance	Laying–Meat	Laying–Breeding	Meat–Breeding
	*p*-Values		
Doxycycline	0.0970	0.4700	0.4730
Vancomycin	<0.0001 *	0.0460 *	0.0300 *
Enrofloxacin	<0.0001 *	0.4560	0.0009 *
^1^ Amoxicillin–clavulanic acid	<0.0001 *	0.0200 *	0.0120 *
Florfenicol	0.0230 *	0.0002 *	<0.0001 *
^2^ Potentiated sulfonamide	0.4420	<0.0001 *	<0.0001 *
Amoxicillin	0.0180 *	0.7410	0.0160 *
Imipenem	0.1910	<0.0001 *	<0.0001 *
Neomycin	<0.0001 *	0.7490	<0.0001 *
Tylosin	<0.0001 *	0.1960	<0.0001 *

* Significant difference (*p* < 0.05); ^1^ 1:2 ratio; ^2^ trimetoprim–sulphametoxazole 1:19 ratio.

**Table 3:** Statistical analysis of resistance by age.

Active Substance	^3^ Young–^4 ^Adult
	*p*-Values
Doxycycline	0.1330
Vancomycin	<0.0001 *
Enrofloxacin	<0.0001 *
^1^ Amoxicillin–clavulanic acid	<0.0001 *
Florfenicol	<0.0001 *
^2^ Potentiated sulfonamide	0.0003 *
Amoxicillin	0.0069 *
Imipenem	0.2570
Neomycin	<0.0001 *
Tylosin	<0.0001 *

* Significant difference (*p* < 0.05); ^1^ 1:2 ratio; ^2^ trimetoprim–sulphametoxazole 1:19 ratio; ^3^ younger than 6 weeks; ^4^ older than 6 weeks.

**Table 4:** Statistical analysis between flock size and resistance rate.

Active Substance	Small–Medium	Small–Large	Medium–Large
	*p*-Values		
Doxycycline	0.1090	<0.0001 *	<0.0001 *
Vancomycin	0.4700	0.2330	0.0841
Enrofloxacin	0.0138 *	0.1840	0.0017 *
^1^ Amoxicillin–clavulanic acid	0.2300	0.0866	0.9250
Florfenicol	0.9130	0.1330	0.2060
^2^ Potentiated sulfonamide	0.6310	0.0028 *	0.0042 *
Amoxicillin	0.5130	0.5400	0.2690
Imipenem	0.7020	0.1840	0.1520
Neomycin	<0.0001 *	0.0003 *	<0.0001 *
Tylosin	<0.0001 *	<0.0001 *	0.5720

* Significant difference (*p* < 0.05); ^1^ 1:2 ratio; ^2^ trimetoprime–sulphametoxazole 1:19 ratio; small—5001–50,000; medium—50,001–100,000; large—>100,001.

**Table 5:** Frequency table of the minimum inhibitory concentration (MIC) values (µg/mL) of the active substances with breakpoints obtained in *Enterococcus* samples of domestic fowl origin (*n* = 499). For each active substance, the top row shows the number of units and the bottom row shows the percentage of each. The red vertical lines indicate the breakpoints.

Antibiotic	BP *	0.001	0.002	0.004	0.008	0.016	0.03	0.06	0.125	0.25	0.5	1	2	4	8	16	32	64	128	256	512	1024	MIC_50_	MIC_90_	^2^ ECOFF
	µg/mL																						µg/mL		
Imipenem	^1^ 16	4	1	6	6	23	12	12	11	13	37	75	107	98	60	26	3	0	0	3	1	1	2	8	4
		0.8%	0.2%	1.2%	1.2%	4.6%	2.4%	2.4%	2.2%	2.6%	7.4%	15.0%	21.4%	19.6%	12.0%	5.2%	0.6%	0.0%	0.0%	0.6%	0.2%	0.2%			
Neomycin	1024										2	8	6	10	26	25	40	59	99	77	53	94	128	1024	256
											0.4%	1.6%	1.2%	2.0%	5.2%	5.0%	8.0%	11.8%	19.8%	15.4%	10.6%	18.8%			
Doxycycline	^1^ 16			3	2	5	1	1	3	11	8	106	30	70	73	70	60	40	13	2	1		8	64	1
				0.6%	0.4%	1.0%	0.2%	0.2%	0.6%	2.2%	1.6%	21.2%	6.0%	14.0%	14.6%	14.0%	12.0%	8.0%	2.6%	0.4%	0.2%				
Florfenicol	^1^ 8										2	5	50	194	135	44	35	12	11	7	3	1	4	32	8
											0.4%	1.0%	10.0%	38.9%	27.1%	8.8%	7.0%	2.4%	2.2%	1.4%	0.6%	0.2%			
Vancomycin	^1^ 32								4	7	30	163	84	37	29	6	2	1	11	43	31	51	2	1024	4
									0.8%	1.4%	6.0%	32.7%	16.8%	7.4%	5.8%	1.2%	0.4%	0.2%	2.2%	8.6%	6.2%	10.2%			
Amoxicillin	^1^ 16	1	0	3	6	9	10	3	10	28	88	116	80	30	11	2	8	4	9	28	29	24	1	512	-
		0.2%	0.0%	0.6%	1.2%	1.8%	2.0%	0.6%	2.0%	5.6%	17.6%	23.2%	16.0%	6.0%	2.2%	0.4%	1.6%	0.8%	1.8%	5.6%	5.8%	4.8%			
^3^ Amoxicillin–clavulanic acid	^1^ 16			2	3	9	13	3	17	35	101	94	72	49	42	19	28	7	5				1	16	-
				0.4%	0.6%	1.8%	2.6%	0.6%	3.4%	7.0%	20.2%	18.8%	14.4%	9.8%	8.4%	3.8%	5.6%	1.4%	1.0%						
Tylosin	8		1	0	0	1	0	0	0	2	7	122	65	29	6	4	6	6	15	28	68	139	128	1024	-
			0.2%	0.0%	0.0%	0.2%	0.0%	0.0%	0.0%	0.4%	1.4%	24.4%	13.0%	5.8%	1.2%	0.8%	1.2%	1.2%	3.0%	5.6%	13.6%	27.9%			
Enrofloxacin	^1^ 4				1	2	1	3	2	22	73	104	62	45	57	40	21	23	16	11	10	6	2	64	-
					0.2%	0.4%	0.2%	0.6%	0.4%	4.4%	14.6%	20.8%	12.4%	9.0%	11.4%	8.0%	4.2%	4.6%	3.2%	2.2%	2.0%	1.2%			
^4^ Potentiated sulfonamide	^1^ 4						1	0	3	3	5	32	44	52	50	39	16	10	15	13	42	174	4	64	-
							0.2%	0.0%	0.6%	0.6%	1.0%	6.4%	8.8%	10.4%	10.0%	7.8%	3.2%	2.0%	3.0%	2.6%	8.4%	34.9%			

* BP—breakpoint; ^1^ Clinical Laboratory Standard Institute (CLSI); ^2^ epidemiological cut-off value (EUCAST); ^3^ 2:1 ratio; ^4^ trimetoprim–sulphamethoxazole 1:19 ratio.

## Data Availability

The data presented in this study are available from the corresponding author upon reasonable request.

## Supplementary Materials

The following supporting information can be downloaded at https://www.mdpi.com/article/10.3390/antibiotics13121194/s1. Table S1: Distribution of sampling dates and utilization types by region; Table S2: The *p*-values obtained from the statistical test of the degree of drug resistance used in the correlation analysis; Table S3: Frequency table of the minimum inhibitory concentration (MIC) values (µg/mL) for agents without breakpoints in *Enterococcus* samples derived from chickens (n = 499). The top row for each agent shows the count, while the bottom row shows the percentage. Figure S1: Reducing distances within clusters. Figure S2: Qualitative result of the cluster analysis. Figure S3: Gap statistics with reduced cluster count and reduced reference data.
