# Supplementary material for: Antimicrobial Susceptibility Profiles of Commensal Enterococcus spp. Isolates from Chickens in Hungarian Poultry Farms Between 2022 and 2023
Source: Antibiotics (Basel). 2024 Dec 7;13(12):1194. doi: 10.3390/antibiotics13121194 (PMC11672767; doi:10.3390/antibiotics13121194)
Supplement: Supplementary file 1 [file antibiotics-13-01194-s001.zip › antibiotics-3319815-supplementary.pdf]

Supplementary Table S1 Distribution of sampling dates and utilization types by region

Region	Sampling time	Utilization
**Észak-Magyarország**	20.10.2022.	laying
	20.10.2022.	laying
	23.06.2023.	meat
**Észak-Alföld**	23.05.2023.	meat
	23.05.2023.	meat
	23.05.2023.	meat
**Dél-Alföld**	12.02.2022.	laying
	16.02.2022.	meat
	16.02.2022.	meat
**Közép-Magyarország**	06.10.2022.	laying
	24.04.2023.	breeding
	24.04.2023.	meat
**Dél-Dunántúl**	25.03.2022.	meat
	17.04.2023.	meat
	16.05.2023.	laying
**Nyugat-Dunántúl**	19.07.2022.	breeding
	09.05.2023.	breeding
	09.05.2023.	breeding
**Közép-Dunántúl**	28.03.2022.	laying
	04.04.2022.	laying
	04.04.2022.	meat
	19.07.2022.	breeding
	09.05.2023.	breeding

Supplementary Table S2 The p-values obtained from the statistical test of the degree of drug resistance used in the correlation analysis

Antibiotics	Doxycycline	Vancomycin	Enrofloxacin	Amoxicillin-clavulanic acid	Florfenicol	Potentiated sulfonamide	Amoxicillin	Imipenem	Neomycin	Tylosin
Doxycycline		<0.0001	<0.0001	<0.0001	<0.0001	<0.0001	<0.0001	0.0006	0.0572	<0.0001
Vancomycin	<0.0001		<0.0001	<0.0001	<0.0001	0.0247	<0.0001	0.0028	0.0668	<0.0001
Enrofloxacin	<0.0001	<0.0001		<0.0001	0.0285	<0.0001	<0.0001	0.0083	0.6241	<0.0001
[1]Amoxicillin-clavulanic acid	<0.0001	<0.0001	<0.0001		<0.0001	0.1689	<0.0001	0.0010	0.6935	<0.0001
Florfenicol	<0.0001	<0.0001	0.0285	<0.0001		0.8252	<0.0001	0.7503	0.0581	<0.0001
[2]Potentiated sulfonamide	<0.0001	0.0247	<0.0001	0.1689	0.8252		0.0078	0.0020	<0.0001	0.6477
Amoxicillin	<0.0001	<0.0001	<0.0001	<0.0001	<0.0001	0.0078		0.0005	0.1155	<0.0001
Imipenem	0.0006	0.0028	0.0083	0.0010	0.7503	0.0020	0.0005		0.5242	0.3798
Neomycin	0.0572	0.0668	0.6241	0.6935	0.0581	<0.0001	0.1155	0.5242		0.0033
Tylosin	<0.0001	<0.0001	<0.0001	<0.0001	<0.0001	0.6477	<0.0001	0.3798	0.0033	

[1]1:2 ratio; [2]trimetoprim-sulphametoxazole 1:19 ration

Supplementary Figure S1 Reducing distances within clusters

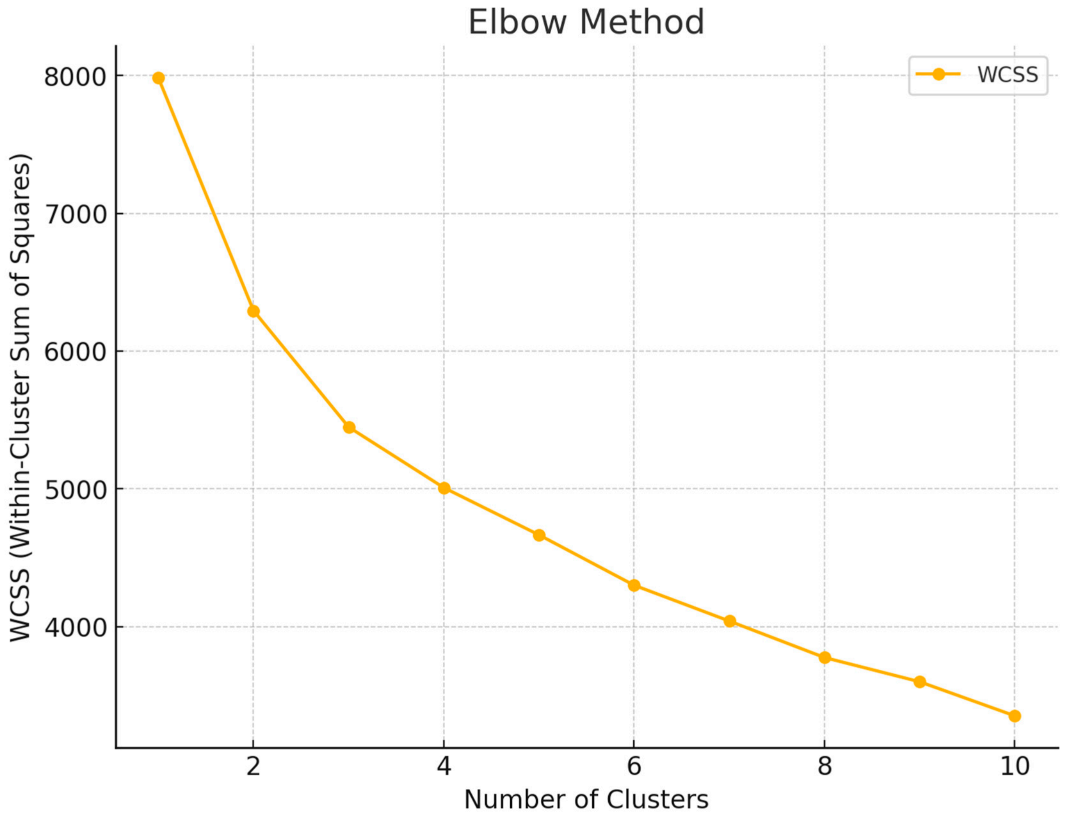

Supplementary Figure S2 Qualitative result of the cluster analysis

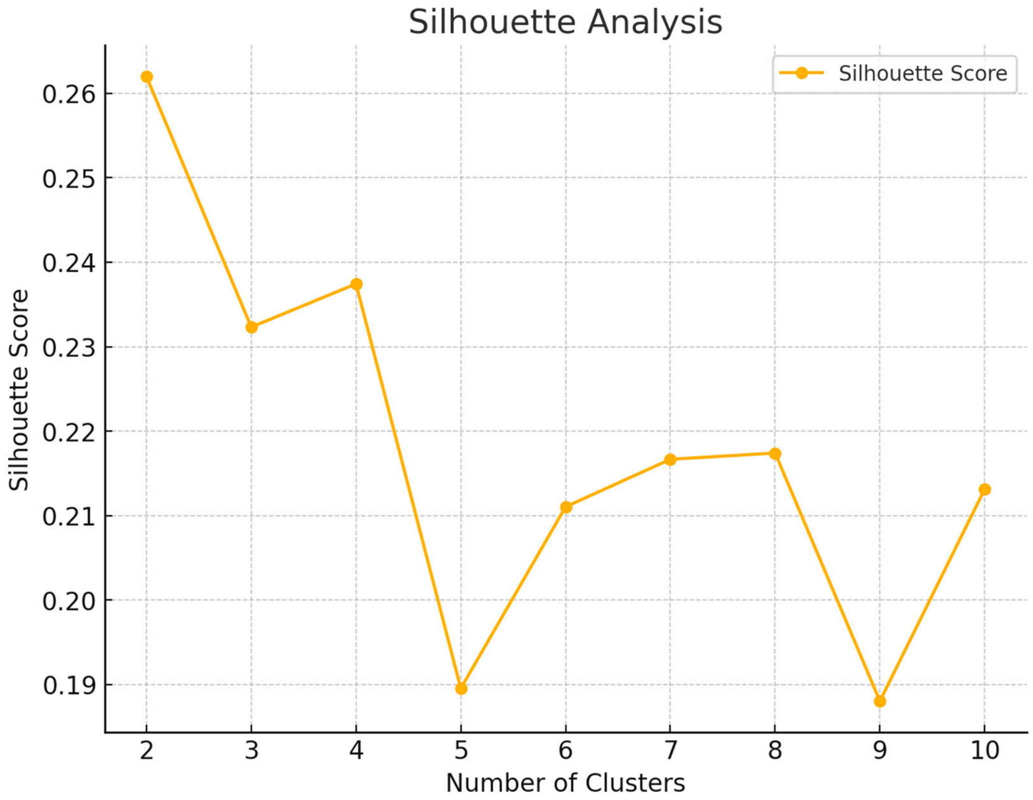

Supplementary Figure S3 Gap statistics with reduced cluster count and reduced reference data

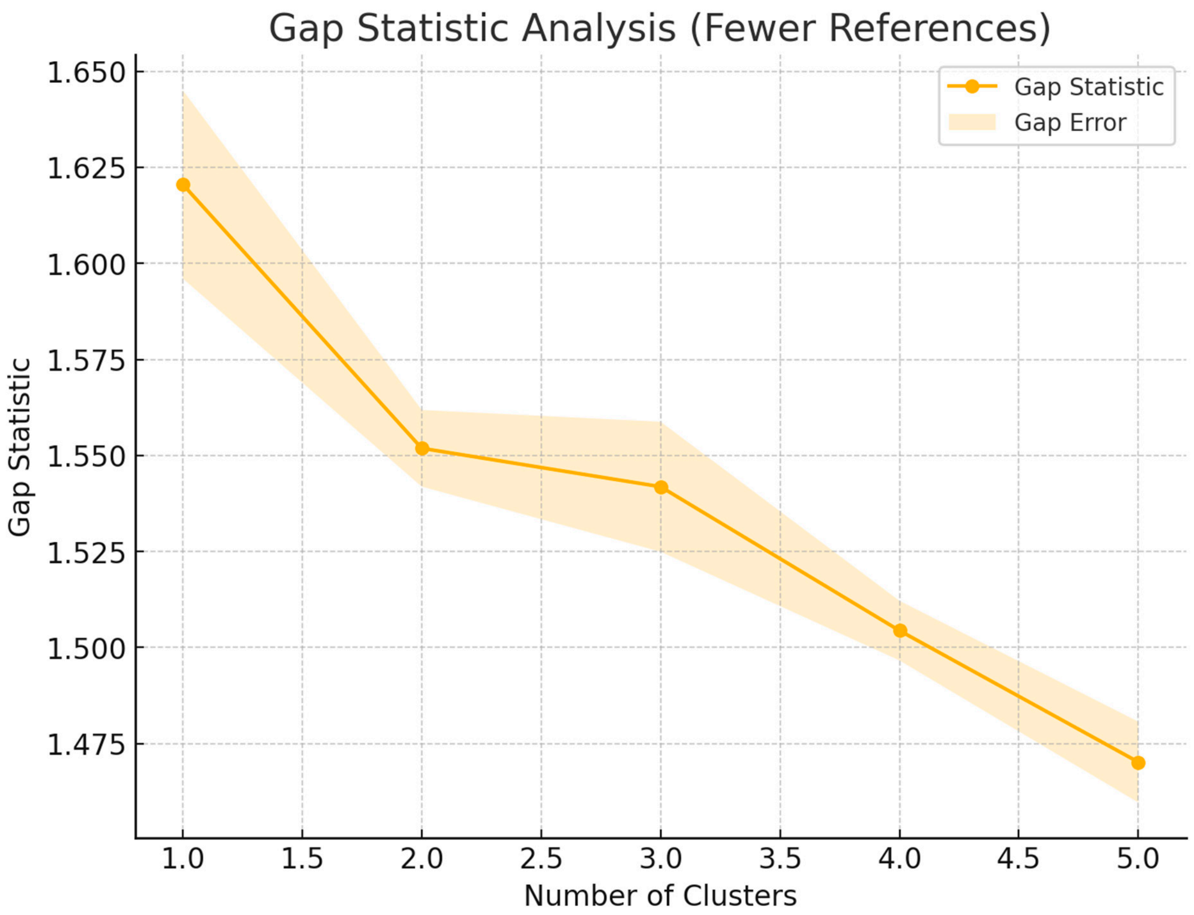

Supplementary Table S3 Frequency table of the minimum inhibitory concentration (MIC) values (µg/mL) for agents without breakpoints in *Enterococcus* samples derived from chickens (*n*=499). The top row for each agent shows the count, while the bottom row shows the percentage.

Antibiotic	0.001	0.002	0.004	0.008	0.016	0.03	0.06	0.125	0.25	0.5	1	2	4	8	16	32	64	128	256	512	1024	MIC$_{50}$	MIC$_{90}$
																						µg/mL	
**Ceftriaxone**					1	9	10	8	8	21	15	6	20	24	27	15	24	44	65	102	100	256	1024
					0.2%	1.8%	2.0%	1.6%	1.6%	4.2%	3.0%	1.2%	4.0%	4.8%	5.4%	3.0%	4.8%	8.8%	13.0%	20.4%	20.0%		
**Spectinomycin**														1	12	53	143	108	69	45	68	128	1024
														0.2%	2.4%	10.6%	28.7%	21.6%	13.8%	9.0%	13.6%		
**Tiamulin**										2	8	11	3	3	22	25	43	106	92	115	69	256	1024
										0.4%	1.6%	2.2%	0.6%	0.6%	4.4%	5.0%	8.6%	21.2%	18.4%	23.0%	13.8%		
**Lincomycin**										3	19	18	27	14	35	46	41	26	28	77	165	256	1024
										0.6%	3.8%	3.6%	5.4%	2.8%	7.0%	9.2%	8.2%	5.2%	5.6%	15.4%	33.1%		
**Colistin**														21	19	9	22	24	37	101	266	1024	1024
														4.2%	3.8%	1.8%	4.4%	4.8%	7.4%	20.2%	53.3%		
